# Structural Changes in Metallic Glass-Forming Liquids on Cooling and Subsequent Vitrification in Relationship with Their Properties

**DOI:** 10.3390/ma15207285

**Published:** 2022-10-18

**Authors:** D. V. Louzguine-Luzgin

**Affiliations:** 1 Advanced Institute for Materials Research (WPI-AIMR), Tohoku University, Aoba-Ku, Sendai 980-8577, Japan; dml@wpi-aimr.tohoku.ac.jp; 2 MathAM-OIL, National Institute of Advanced Industrial Science and Technology (AIST), Sendai 980-8577, Japan

**Keywords:** metallic glass, liquid, structure, fragility, viscosity

## Abstract

The present review is related to the studies of structural changes observed in metallic glass-forming liquids on cooling and subsequent vitrification in terms of radial distribution function and its analogues. These structural changes are discussed in relationship with liquid’s properties, especially the relaxation time and viscosity. These changes are found to be directly responsible for liquid fragility: deviation of the temperature dependence of viscosity of a supercooled liquid from the Arrhenius equation through modification of the activation energy for viscous flow. Further studies of this phenomenon are necessary to provide direct mathematical correlation between the atomic structure and properties.

## 1. Introduction

Although metallic amorphous thin films have been produced since the middle of the past century and slightly earlier [1] active research on metallic glasses started since a pioneering work on a Au-Si alloy produced by rapid solidification in 1960 [2]. Different production methods are used depending on the glass-forming ability of the material. Apart from thin films [3] amorphous pure metals (mostly BCC metals), can be prepared by a discharge in small spheres [4] while marginal glass-formers are produced by rapid (compared to conventional metallurgical methods) solidification from a liquid phase. Bulk glassy alloys or bulk metallic glasses can be defined as 3-dimentional volumetric glassy articles with a size of not less than 1 mm in every spatial dimension (10 mm by other definition). They are produced in the thickness range of 1–100 mm by various casting processes [5,6]. Pd- and Zr-based BMGs are among the best metallic glass-formers known to date [7,8] with good glass-forming ability and thermal stability of the supercooled liquid [9]. Bulk metallic glasses are industrially important owing to their excellent physical [10] mechanical [6], magnetic [11] and other properties [12]. They are originally formed in ternary, quaternary and later even in binary alloys [12,13]. Recently, there has been a steady interest in multicomponent bulk metallic glassy alloys [14], including those without a base component [15]. Glass/crystal composites are also important structural and functional materials [16]. 

Structure of liquids and glasses was studied by conventional X-ray diffraction (small angle and wide angle methods) [17,18], synchrotron radiation X-ray experiments [19,20] neutron diffraction [21] (especially useful for light elements like B, Si, C, P, etc.), high-resolution transmission electron microscopy [13,22] fluctuation electron microscopy (FEM) [23] and other methods. Although it is very hard to achieve for conductive materials, ultra-high vacuum scanning tunneling microscopy was found to attain atomic scale resolution in metallic glassy surface images [24]. Moreover, it was found to present not only the atomic-scale surface topology but showed only one kind of atomic species (either Ni or Nb) depending on the applied bias (either negative or positive) owing to the difference in the partial electronic density of states.

Although the structure of metallic glasses and liquids is disordered, one can define the degree of topological (TSRO) and chemical (CSRO) short-range order. For example, metalloid-centered clusters were observed in Ni-P metal-metalloid metallic glasses [25]. Structure of metallic glasses has been described by different models [26,27]. At the same time the structure of oxide glasses is significantly different from that of metallic glasses [28]. 

Although the structure of liquids changes only slightly on supercooling [29] as it will be shown below, liquids continuously change their atomic arrangements on cooling. It is reflected, for example, in thermal expansion in the first coordination shell on cooling compared to a contraction in the other shells and local structure changes [13]. Then there is an important question is there a connection between these structural changes with temperature on cooling towards glass-transition and dynamics of liquids. The main purpose of this article is to show the effect of structural changes in the melts of metallic glass-forming alloys during cooling on the change in the activation energy of viscous flow, and accordingly, the fragility of these liquids. 

## 2. Structural Changes in Supercooled Liquids and Glass-Transition Process

**Figure 1:**
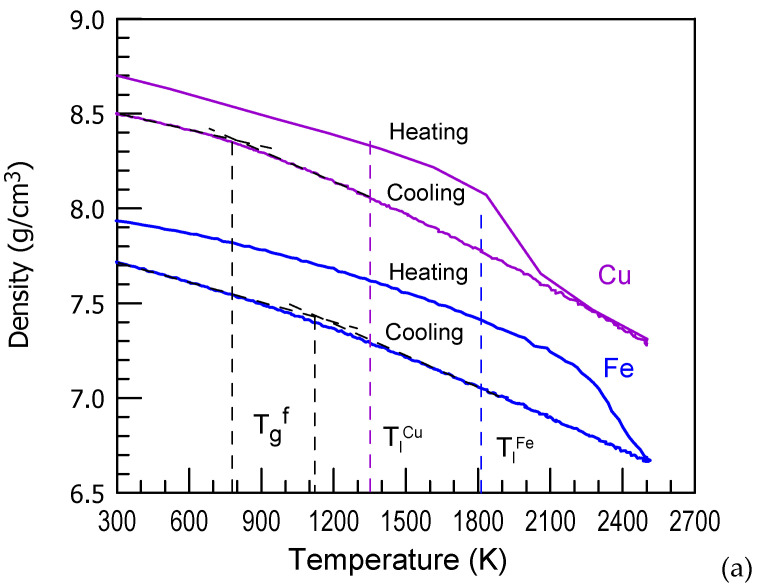

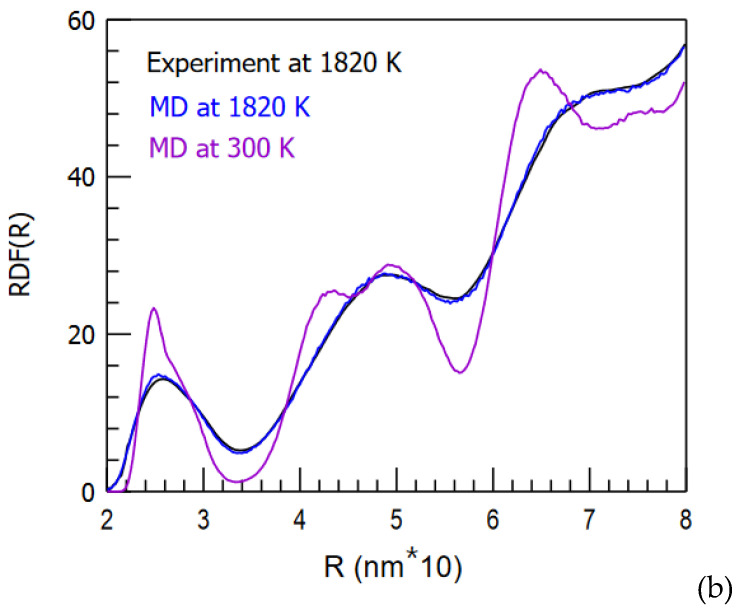
Density changes on heating and cooling of Cu and Fe as a function temperature (**a**). Reproduced from [36] with permission of MDPI. *RDFs* of Fe in liquid at 1820 K (experiment and MD simulation) and glassy at 300 K (MD simulation only) state (**b**). The experimental *RDF* curve is taken from [37].

Liquids are different from solids because they have a zero value of low frequency shear modulus [30]. As below the liquidus (*T_l_*) and solidus (*T_s_*) temperatures thermodynamically equilibrium phase is a crystalline one(s), a liquid supercooled below these temperatures is in a metastable state. Although in its metastable supercooled state at a certain temperature it can be relaxed and exist for a certain period of time without structural changes [29,31], as will be mentioned below, its structure is already somewhat different from that above the liquidus/solidus temperatures. Moreover, if its glass-forming ability is high enough it can be further supercooled and form a glassy phase. The process of glass-transition or vitrification is connected with solidification of a liquid phase on cooling without its crystallization [32,33]. Density (*ρ*) of a liquid phase changes faster with temperature compared to that of a competing crystalline one [34,35]. The glass-transition region is connected with the change in d*ρ*/dT or other properties from a relatively high value (liquid phase) to a lower value (glassy phase) as shown in Figure 1a [36]. Such a change of the thermal expansion coefficient on glass-transition was clearly demonstrated for alloys too. The resulted structures are characterized by much broader peaks in the radial distribution functions (*RDFs(R)*) compared to those of crystals (Figure 1b) [36,37] because liquids/glasses rather scatter X-rays while on crystals they undergo lattice diffraction. *RDF(R)* indicates average probability of finding an atom at a radial distance (*R*) from an arbitrarily chosen atom.

Vitrification of metallic liquids illustrated in Figure 1a was modeled using molecular dynamics (MD) simulation. High critical cooling rates of about 10^13^ K/s are required to vitrify pure metals in MD simulation [38,39] though theoretical calculations [40] and experiments with submicron scale samples [4] suggest a lower critical cooling rate (of about 10^9^ K/s) for glass-transition in pure metals, especially with BCC lattice. Formation of an alloy by adding second component to a pure metal, for example Zr to Cu, drastically decreases the critical cooling rate [41] but does not affect the glass-transition region. 

**Figure 2:**
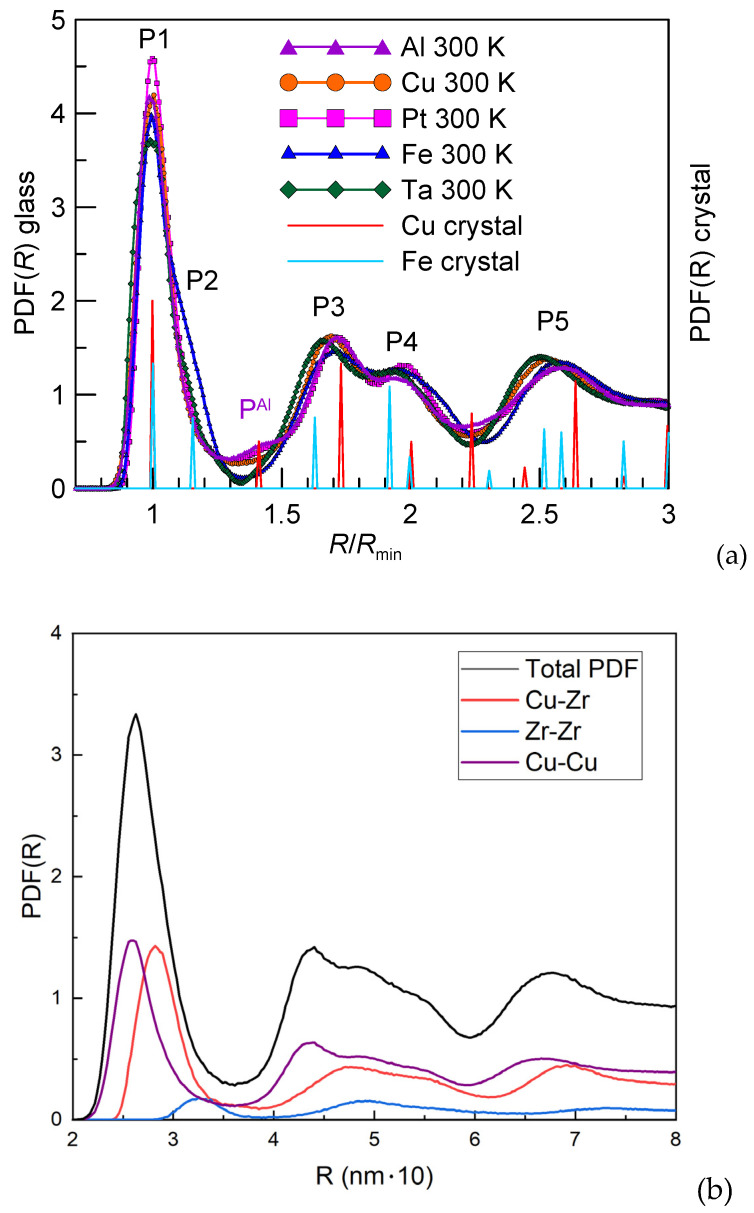
(**a**) *PDF(R)*_glass_ of glassy Al, Cu, Fe, Pt and Ta as a function of radial distance (*R*) divided by the nearest interatomic distance (*R_min_*) in the corresponding crystal on the left side Y axis and *PDF(R)*_crystal_ of the corresponding crystal: Cu as a representative of FCC one and Fe of a BCC one on the right side Y axis. (**b**) Partial and total *PDF(R)*s of the Cu_80_Zr_20_ alloy cooled at 10^12^ K/s (MD simulation).

The transition from liquid to glass can also be detected using temperature variation in the first maximum and minimum of *RDF*(*R*) or *PDF*(*R*) [42,43]. The reduced radial distribution function or pair distribution function (*PDF*(*R*), also called *g*(*R*)) corresponds to the number of atoms at a distance *R* from an arbitrarily chosen atom divided by an average number of atoms geometrically expected at this distance from the atomic number density. *PDF(R)* of some FCC and BCC metals obtained using classical molecular dynamics simulation on cooling at 10^13^ K/s to room temperature are shown in Figure 2a in comparison with those of crystals. They clearly show TSRO in pure metals [44] The metals used given together with sources of the interatomic potentials are: FCC-type (Al and Cu [45] as well as Pt [46]), 108,000 atoms and BCC-type metals (Fe [47] and Ta [48]), 128,000 atoms. The details of computational procedure can be found in [36]. *PDF(R)* is plotted as a function of distance (*R*) divided by the minimum interatomic distance (*R_min_*) in the corresponding crystal (either FCC or BCC).

The coordination numbers in the first coordination shell in liquid derived from the radial distribution functions shown in Figure 1b for Fe are 12.6 and 12.5, respectively, for experiment and MD simulation. The coordination number of 13.3 is obtained in the glassy state at 300 K (MD simulation). It is slightly lower than 14 atoms (8 + 4) in BCC crystalline state corresponding to the distances in the first coordination shell of glass (Figure 2a). The atomic number density obtained from MD simulation was 0.076 and 0.083 at/A^3^ in liquid and glassy states, respectively. 

The points of change of d*ρ*/d*T* slope are 1100 K for Fe and 800 K for Cu (Figure 1a). For Fe it corresponds well to a predicted one when volume of the liquid becomes similar to that of the competing crystalline phase [49]. In some glass-forming alloys, such as Cu_50_Zr_50_/(Cu_50_Zr_50_)_95_Al_5_ [50] and Al_86_Ni_4_Co_4_Gd_6_ [51], glass transition takes place in accordance with this criterion. The glass-transition temperature (*T_g_*) of the Al_86_Ni_4_Co_4_Gd_6_ alloy of 550 K measured by DSC on heating at relatively slow heating [52] corresponds quite well to that extrapolated from the density as a function of temperature curves for liquid and crystalline phases [51]. The intersection of the tangents of the volume as a function of temperature curves in liquid and vitreous state of the Pd_43_Cu_27_Ni_10_P_20_ melt suggests volumetric *T*_g_ of 572 K and equivolume (liquid-crystal) temperature is 460 K [53] while vitrification takes place well above it. Moreover, such an equivolume temperature is not attained in the Co_48_Fe_25_Si_4_B_19_Nb_4_ glassy alloy [54]. Vitrification of these alloys is not limited by this criterion. 

As it was mentioned above *T_g_* is usually measured in a calorimeter on heating. *T_g_* as high as 900 K is obtained for Ferrous-metals based metallic glasses [55] but for Ca-Li-based alloys it is as low as 308 K [56]. There is also a concept of fictive glass transition temperature calculated from the heat overshoot in differential calorimetry measurements [57]. An arbitrary *T_g_* is defined as a temperature at which the dynamic viscosity of a liquid/melt reaches 10^12^ Pa⋅s on cooling. For some materials 10^12^ Pa⋅s belongs to the calorimetrically detected glass-transition region though but not for all substances [58]. The equilibrium viscosity at calorimetrically determined *T*_g_ was found to range from about 10^10^ to 10^12^ Pa∙s [59]. The non-equilibrium viscosity at a Non-Newtonian flow under high enough stress can be as low as 10^17^ Pa⋅s even at room temperature [60]. Bartenev–Ritland phenomenological equation [29] links *T*_g_ with the cooling rate (*β*) as:

1/*T*_g_ = *a* − *b*∙ln(*β*) (1)

where *a* and *b* are the constants. It indicates a weak, logarithmic temperature dependence of *T*_g_ from *β*.

Five broad *PDF*(*R*) peaks (some of which are shoulders of one peak) corresponding to first (P1 and P2), second (P3 and P4) and third (P5) coordination shells are marked in Figure 2a. It can be seen that all these metals somehow inherit their local crystalline order though no traces of a crystalline phase are found except for Al in which crystal nucleation took place and 1.5% of the volume fraction of FCC phase was detected. It is manifested by an intermediate peak P^Al^ in between the first and second coordination shells. BCC metals, especially Fe, show strong P2 peak corresponding to the second peak of BCC crystalline phase and deep *PDF*(*R*) minimum between the first and second coordination shells while in case of FCC-type glassy metal Cu the minimum (0.26) is about 2.5 times larger than that of Fe (0.11) and 4 times larger than that of Ta (0.06). This indicates extremely small number of atoms in BCC-type glassy metals between two coordination shells at 1.35 *R/R_min_*. In other words the first and second coordination shells are very well separated in accordance with absence of atoms at 1.2–1.5 of *R/R_min_* in BCC crystals. The minimum *PDF(R)* value between the first and second coordination shells of FCC-type metal Pt has an intermediate value between those of Cu and Al though no crystallization was found for Pt.

P3 and P4 of both FCC- and BCC-type glassy metals related to the second coordination shell of glass correspond quite well to the positions of crystalline peaks between 1.5 and 2.2 of *R/R_min_*. These P3 and P4 are formed on glass-transition from a single peak in liquid as can be seen in Figure 1b. The position of P5 of FCC-type metal glasses poorly corresponds to the crystalline peaks while this peak of BCC-type metal glasses is more reasonably described by the corresponding crystalline structure. In case of alloys, such as a Cu_80_Zr_20_ binary one for example, total *PDF*(*R*) is the sum of three partial *PDF*(*R*)s as shown in Figure 2b. This alloy was modeled with the following potential [61]. Atomic arrangements in alloys also create CSRO.

**Figure 3:**
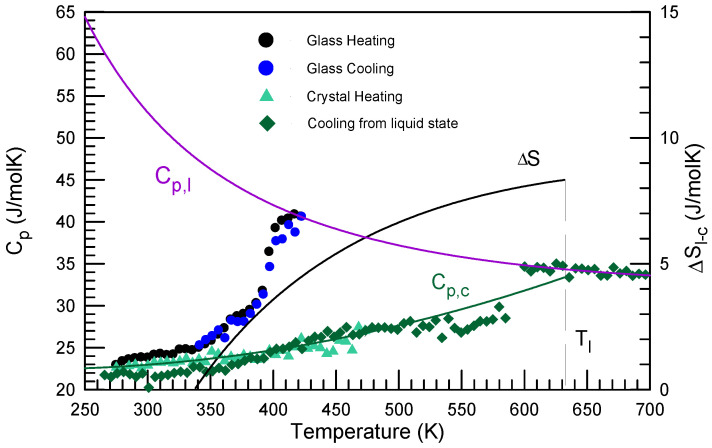
*C_p_* of the Au_49_Cu_26.9_Ag_5.5_Pd_2.3_Si_16.3_ alloy in liquid (l), glassy and crystalline (c) state as a function of temperature (left Y axis) and the entropy difference between the liquid and crystalline state Δ*S*. The Kauzmann temperature is 340 K. Reproduced from [64] with permission of Elsevier.

In calorimetry the glass-transition phenomenon is characterized by the specific heat capacity (*C_p_*) change. It changes on heating from a relatively low value typical for a glassy phase (close to 3R) to about 1.5 times higher one typical for a liquid one. In some alloys variation of *C_p_* in the glass-transition region suggests double-stage behavior [62,63]. For example, Au_49_Cu_26.9_Ag_5.5_Pd_2.3_Si_16.3_ bulk metallic glass showed two different slopes indicating two glass-transition processes one starting at a low temperature around 340 K another at 380 K as one can observe in Figure 3 [64]. It is likely related to the different diffusion coefficients of the constituent elements in this alloy. A well-known Kauzmann’s temperature (*T_K_*) [65] connected with zero entropy difference (Δ*S*) between the liquid and crystalline phases at *T_K_* is also illustrated in Figure 3. Similar results were obtained in Ref. [66].

It was also suggested that a configuron (broken bond) phase forms in amorphous silica above *T_g_* indicating that the glass transition can be a second-order phase transformation. It is suggested to take place differently on heating and on cooling through the glass–liquid transition region [67]. 

## 3. Liquid Viscosity and Beginning of Non-Arrhenius Type Temperature Dependence on Cooling

As well as crystals liquids retain their volume but flow under gravity [68,69]. Owing to thermal excitations their local atomic structure constantly undergoes some changes even at a constant temperature [70,71]. Equilibrium metallic melts show nearly Arrhenius-type temperature dependence of the dynamic viscosity (*η*):

*η* = *η*_0_*exp(E_a_/RT)* (2)

where *η_0_* is a pre-exponential factor, here *R* is the gas constant (please note that it is different from the radial distance defined above) and *E_a_* is an activation energy for viscous flow (some values are listed in Refs. [35,72]). Viscosity is related to the relaxation time [73]. 

There is a more precise equation for the temperature dependence of *η* derived in 30th of the past century from the free volume theory [74] with *η_0_* as a temperature dependent term:

*η* = (*d*/*V_m_*)⋅(2π*MRT*)^1/2^⋅*exp*(*E_a_*/*RT*) (3)

for mol of atoms where *d^3^* is free space, *V_m_* is molar volume, *M* is molar mass. It was later reorganized using both the free volume and the activation energy approaches into the following form [75]:

*η* = *A*⋅(*MT*)^1/2^/*V_m_*^2/3^⋅*exp*(*B*⋅*T_m_/T*) (4)

where *A* and *B* are constants and *T_m_* is the melting temperature making *E_a_* dependent on *T_m_*. It was shown to describe well temperature dependence of viscosity of the most of metals with the values of *A* and *B* close to 1.80 ± 0.39 × 10^–8^ (J/Kmol^1/3^)^1/2^ and 2.34 ± 0.20, respectively. Equations (3) and (4) indicate a slight deviation from linearity of ln(*η*) versus 1/*T* plot which will cause some interesting high-temperature effects to be discussed below.

However, on cooling below a certain temperature, in some cases called crossover temperature (*T_A_*), which is considered to be slightly above the liquidus temperature (*T_l_*) for metallic glasses [76], liquids/melts exhibit a non-Arrhenius temperature dependence on viscosity known as fragility of liquids [70,77] to be discussed below. Although a slight departure from the Arrhenius temperature dependence of viscosity takes place above *T_l_* most significant changes occur in the supercooled liquid region.

Such a change from the Arrhenius temperature dependence of viscosity of glass-forming liquids to non-Arrhenius one is detected using viscosity [78], relaxation time [79] or other values. A crossover temperature of about 870 K found for the Au_50_Cu_25.5_Ag_7.5_Si_17_ glass-forming liquid was detected by a nuclear magnetic resonance (NMR) device while the liquidus and the glass transition temperatures are 663 K and 378 K, respectively [80]. This alloy shows a very high *T*_A_/*T*_l_ ratio of 1.31. A correlation obtained between *T_A_* and *T_g_* was found to suggest that the cooperative atomic processes leading to the glass transition are also characteristic of the equilibrium liquid [76]. It was also suggested that deviation from the Arrhenius temperature dependence of viscosity is related to the onset of local inhomogeneities in the glass forming materials on cooling due to cooperative processes [81]. On the other hand separation of the diffusion coefficients of different elements in liquids takes place below *T_l_* [82]. 

**Figure 4:**
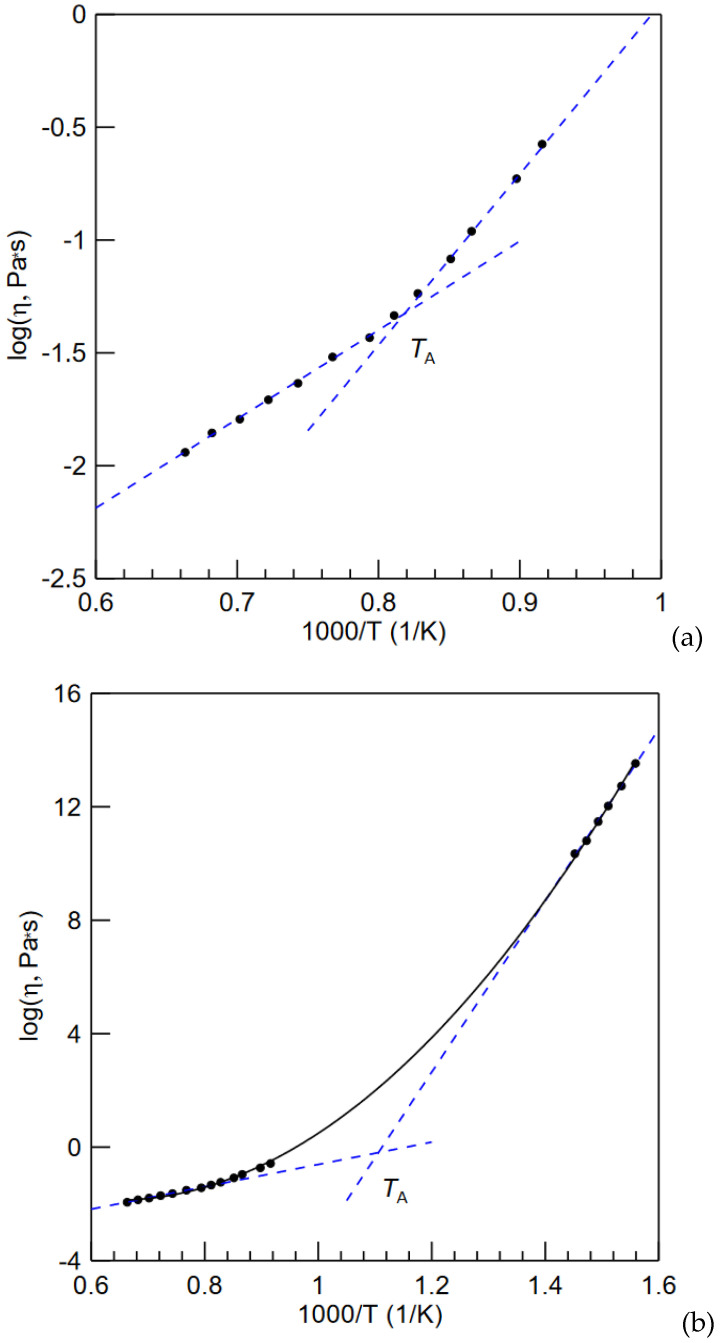
Log(*η*) of the Zr_58.5_Cu_15.6_Ni_12.8_Al_10.3_Nb_2.8_ (Vit106a) alloy as a function of 1000/T in two ranges (**a**,**b**). The data points are obtained from Ref. [76].

Here one should note that there are two main methods in detecting the change of slope of any value including *T_A_*: by slightest deviation of ln(*η*) versus 1/*T* plot from the linearity or by applying two tangents below and above the deflection point. The former method suggests *T_A_*/*T_g_* ratio of about 2 for metallic liquids [76] and about 1 for fragile molecular liquids [83]. The latter one sets *T_A_* closer to *T_l_*. For example, from the viscosity data for Zr_58.5_Cu_15.6_Ni_12.8_Al_10.3_Nb_2.8_ (Vit106a) BMG forming liquid plotted in Figure 4a [76] by two tangents one can obtain *T_A_* = 1219 K. The reported values of *T_A_*, and *T_g_* are 1360 and 672 K, respectively, (or 1276 and 668 K, respectively, from Ref. [84]) while *T_l_* is 832 °C or 1105 K [85]. If both high temperature and low temperature limits of the viscosity slope are used then the estimated *T_A_* is only 909 K, which is below *T_l_* (Figure 4b). 

Nevertheless, if one accepts that *T_A_* is about 1.2*T_l_* then the question is why the crossover occurs in equilibrium liquid state. The question of whether a molten alloy liquid is homogeneous on the atomic scale above its liquidus temperature or there are some clusters has been a subject of discussion for many years. Some experimental studies suggest that atomic scale heterogeneities exist in the equilibrium liquid alloys especially at eutectic compositions suitable for glass formation [86,87]. This may explain why *T*_A_ is above *T*_l_. However, as it will be shown below, structural changes in liquids above the liquidus temperature are hardly detectable by X-ray or neutron diffractometry and mostly take place well below it. 

## 4. Liquid Fragility Concept

**Figure 5:**
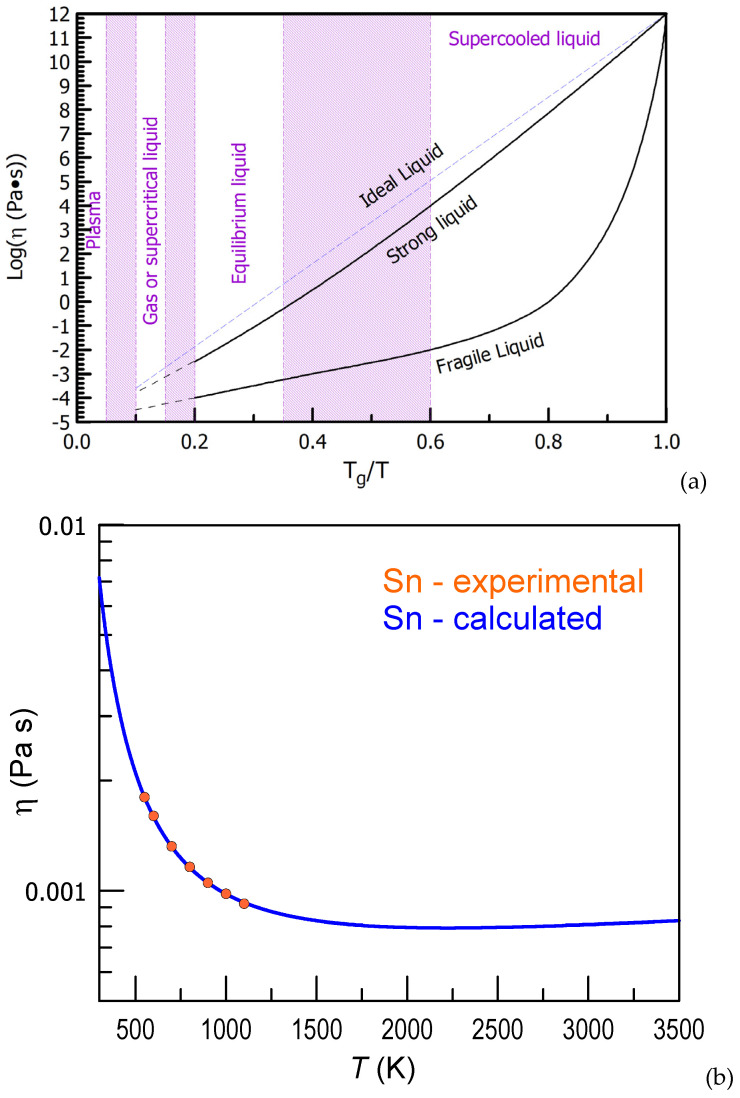
(**a**) A schematic plot of the viscosity as a function of temperature for strong and fragile liquids together with the corresponding phase regions. Filled areas represent the temperature regions of liquidus, boiling and plasma formation temperatures depending on the alloy composition. Reproduced from [13] with permission of Materials Research Forum LLC. (**b**) Temperature dependence of viscosity of Sn calculated by Formula (4) according to Ref. [75] and experimental viscosity values from Ref. [91].

The inverse temperature—logarithmic viscosity plot [88] illustrates the difference between so-called “strong” (following Arrhenius equation) and “fragile” (deviating from that) liquids. The plot scales liquid viscosity by the reduced temperature normalized by *T_g_* as it is schematically shown in Figure 5a [13]. Strong and fragile liquids exhibit different degrees of deviation from the Arrhenius temperature dependence (Equation (2)).

Here one should mention that temperature dependence of viscosity in the high-temperature region is different from that near *T*_m_ [89,90]. As predicted by Equations (3) and (4) the square root of temperature term, being marginal at low temperature, starts to dominate over the exponential decay term at high temperature leading to rise of viscosity in the supercritical liquid region. For example, the temperature dependence of viscosity of liquid Sn calculated according to the values given in Ref. [75] is shown in Figure 5b (provided that boiling expected at 2875 K is suppressed by supercritical pressure) together with some experimental points from Ref. [91]. Such dependence is also observed in the case of many molecular substances (water, methanol and ethanol) under supercritical pressure [92].

Also, boiling of liquid must be avoided by applying an overcritical external pressure [89]. The high-temperature part of the viscosity plot cannot exist below the critical point on pressure—temperature phase diagram. Above this point a liquid/gaseous phase can reach high temperatures without undergoing boiling but it inevitably transforms to plasma at extremely high temperatures. Rise of viscosity of supercritical fluids with temperature was predicted [93] and observed before [94]. Here one should also mention that although a non-zero external pressure value must be applied for a liquid to be thermodynamically stable versus gaseous phase upon MD simulation liquids are often modeled at zero pressure. The fact that such liquids do not evaporate is connected with a relatively short simulation time, usually up to tens of nanoseconds, at present and the gaseous phase has no time to nucleate. 

Stronger liquids, in general, have a higher viscosity in the entire temperature range from *T_l_* and *T_g_* which is favorable for improving the glass-forming ability [95,96]. For example ZrO_2_ was found to be extremely fragile liquid in line with its low glass-forming ability [97]. However, it is suggested that fragile liquids have a lower Gibbs free energy difference between the liquid and crystalline phases, and thus, a lower driving force to crystallization [98]. Accordingly, the authors of Ref. [99] classify BMGs into thermodynamically stabilized and kinetically stabilized glass-formers. Small driving force for crystallization is typical for thermodynamically stabilized glasses while their fragility can be high. On the other hand, the glass-forming ability of kinetically stabilized glasses increases with decreasing fragility.

The fragility index (*m*) [98,100] of a supercooled liquid (also called kinetic fragility) is calculated as the temperature approaches *T_g_* on cooling as a derivative:

*m* = *dlog(η)/d(T_g_/T)* (5)

There are some results indicating that m can be determined from calorimetrical measurements [101]. 

A famous Vogel-Fulcher-Tammann-Hesse equation [89]:

*η* = *η*_0_*exp[D∗T_0_/(T–T_0_)]* (6)

where *η_0_*, *D* (as an indicator of the fragility) and *T_0_* are the fitting parameters is another one used for this purpose but describes the temperature dependence of viscosity well only in an intermediate temperature region [102]. Other equations which have a larger number of fitting parameters make a better representation of the entire viscosity plot [103,104]. It was also shown, that it is possible to reproduce the experimental viscosity data using only two fitting parameters [105].

Fragility also can be expressed by the ratio (*R*_D_) of the activation energies for viscous flow in the Equation (2). One can use this ratio of the energies: high (*E*_H_) value, at low (close to *T_g_*) and low (*E*_L_) value at high temperature region (above *T_l_*) [106,107]:

*R*_D_ = *E*_H_/*E*_L_ (7)

More generally the universal temperature relationship for the activation energy of viscous flow of liquids is [108]:

*E(T)* = *E_L_* + *RT∙ln[1 + exp(−S_d_/R)∙exp((E_H_ − E_L_)/RT)]* (8)

which depends on these two asymptotic energies and on the entropy of configurons *S_d_*. Also, spatial heterogeneities (soft and hard zones) can alter the dynamics of supercooled liquids and glasses [109,110].

Smaller *m* or larger *D* values result in smaller deviation from the Arrhenius temperature dependence of viscosity Equation (2). Typical values of the parameter *m* are shown in Table 1 [111,112]. Among alloys Pd-, Pt-, and Ni-based liquid alloys, in general, are more fragile than the Mg-based and Zr-based ones while oxides like SiO_2_ and GeO_2_ are among the strongest glass-formers. Molecular liquids are usually fragile with some unusual exceptions.

Various ionic liquids: salts in the liquid state also tend to vitrify on cooling. They range from moderately fragile 1-Methyl-3-octylimidazolium chloride with m = 39 to extremely fragile 1-Butyl-1-methypyrrolidinium bis(trifluoromethylsulfonyl)imide with m = 115. The glass transition temperature, heat capacity change at *T*_g_, and fragility index m of ionic glass-formers were found to be somehow related to the molar mass (M) of a material [113]. 

A nonergodicity factor at low temperatures is related to the vibrational properties of a glass and to the curvature of the energy minimum. Temperature dependence of a nonergodicity factor related to the vibrational dynamics of a glass at low temperatures is also found to be related to the fragility of a glass-forming liquid [114]. The ratio of the instantaneous shear and bulk moduli is also found to determine dynamics of glass-forming liquids through the activation energy of the structural relaxation, and thus liquid fragility [115]. Knowledge of liquid viscosity and fragility is important for determination of its formability under stress [116].

Thermodynamic fragility [117] is related to the entropy difference between the liquid (*S_l_*) and the competing crystalline phase(s) (*S_c_*) (see Figure 3):

Δ*S* = *S_l_* − *S_c_* (9)

provided that the vibrational entropies of the liquid and crystal are equal which may be not so. There is a relationship between the viscosity and the configurational entropy (*S_Con_*),

*η* = *η*_0_
*exp[C/(T∙S_Con_)]* (10)

where, *C* is a constant and *S_Con_* is the configurational part of the entropy at a certain temperature. It was suggested that non-Arrhenius behavior of fragile supercooled liquids can be related to an increase in cooperative relaxation at low temperatures decreasing the minimum number of particles that rearrange on an elementary relaxation event. The number of particles in a cooperatively rearranging region can be inversely proportional to the configurational entropy. 

**Figure 6:**
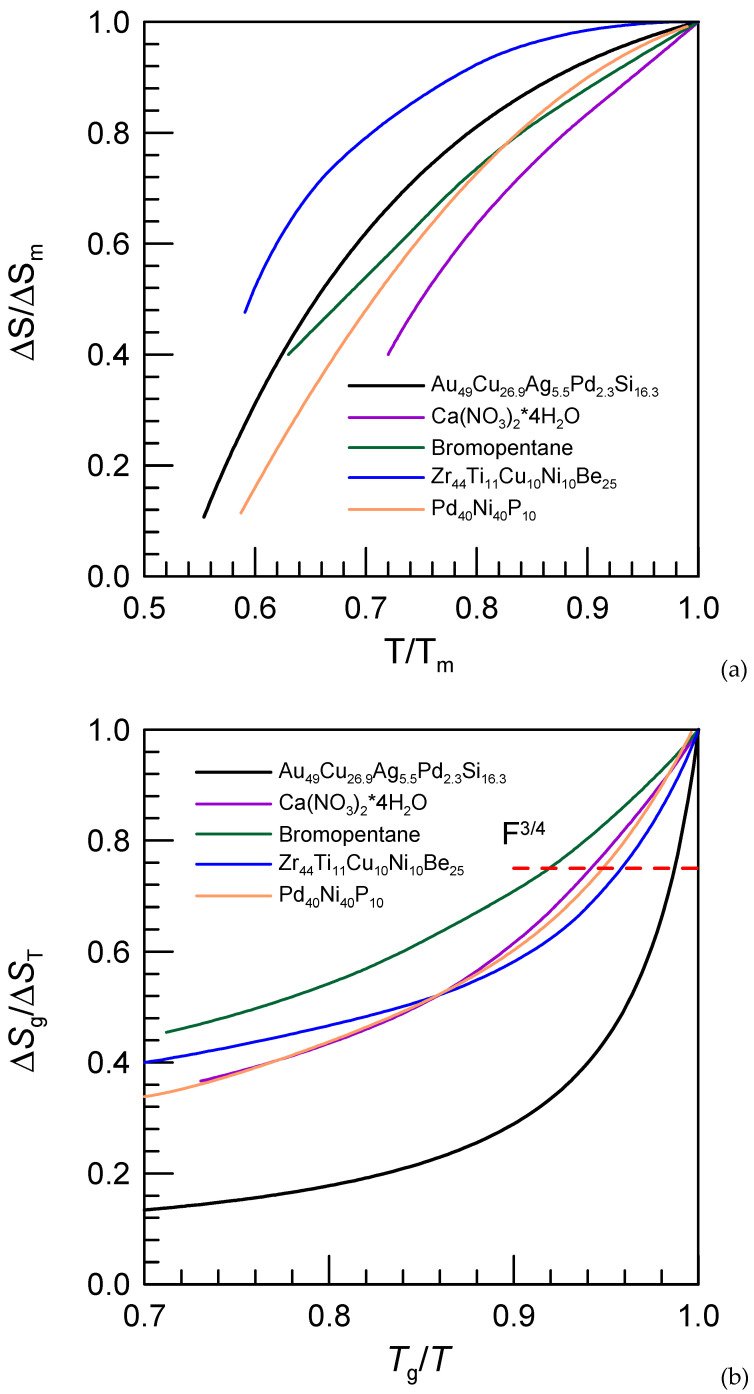
Δ*S*(*T*)/Δ*S*(*T_m_*) as a function of *T*/*T_m_* (**a**) and Δ*S*(*T*_g_)/Δ*S*(*T*) as a function of *T_g_*/*T* (**b**) for different materials, as indicated.

The plots of Δ*S*(*T*)/Δ*S*(*T_m_*) as a function of *T*/*T_m_* [118] and Δ*S*(*T*_g_)/Δ*S*(*T*) as a function of *T_g_*/*T* [58] are plotted in Figure 6. The data for Ca(NO_3_)_2_∙4H_2_O [119], 3-bromopentane [120] Zr_44_Ti_11_Cu_10_Ni_10_Be_25_ [121] and Pd_40_Ni_40_P_20_ [122] are shown. One can see that at a high temperature variation of Δ*S*(*T*)/Δ*S*(*T_m_*) as a function of *T*/*T*_m_ for the Zr_44_Ti_11_Cu_10_Ni_10_Be_25_ and Au_49_Cu_26.9_Ag_5.5_Pd_2.3_Si_16.3_ alloys is much more shallow than that of other glass-forming liquids. Oppositely the Au_49_Cu_26.9_Ag_5.5_Pd_2.3_Si_16.3_ glass-forming alloy has one of the steepest variations of Δ*S*(*T*_g_)/Δ*S*(*T*) as a function of *T_g_*/*T* (Figure 6b) and extremely high *F*^3/4^ thermodynamic fragility value of 0.99. It is in line with relatively high kinetic fragility of this alloy with *m* = 52.8 [123] (*m* = 46 in Table 1).

The excess entropy in metallic glasses is dominated by the configurational entropy while the excess vibrational entropy was found to be very small [124]. To the contrary molecular and network glasses indicate vibrational contribution to the excess liquid/glass entropy [125].

Liquid-liquid transitions in deeply supercooled state [126] take place in some substances. They have been reported to take place in water [127] and other substances as a function of pressure. However, they are suggested to happen even at ambient pressure. Anomalous variation in a liquid viscosity at ambient pressure was observed for some glass-forming alloys including Fe- [128], Yb-Zn [129], Zr-based bulk glass-forming alloys [130,131] and other transition-metal-based alloys [132]. These processes are insufficiently studied and require further investigation because they definitely influence the properties such as viscosity. The structural changes at such processes are only slightly studied and require more attention. 

## 5. Structural Origin of Liquid Fragility

Statistically averaged liquid structures are nearly constant above *T*_l_ (and *T*_A_) but below they start changing rapidly with temperature when it becomes close to *T*_g_, especially for relatively fragile metallic glasses. Container-less aerodynamic levitation of the sample heated by laser [133] in an inert gas is usually used to heat up, melt and cool down the metallic glasses in-situ under X-ray radiation. In some cases the sample is placed in a silica container and heated up by an induction coil. The diffraction intensities shall be recorded during cooling and vitrification with a high enough time resolution. In-situ X-ray diffraction experiments are usually carried out using synchrotron radiation because high beam intensity is required for quick spectra recording. After necessary corrections the total structure factor *S(Q)* and the interference function *Qi(Q)* where *Q* is the scattering vector are obtained. The radial distribution *RDF(R)* and pair distribution functions *PDF*(R)/g(*R*) and *G(R)* are obtained by the Fourier transform of *S(Q)* or *Qi(Q)*. As the noise level at high *Q* values is high the Fourier transform is usually performed until *Q*~150 nm^–1^ though higher *Q* values are preferred. 

Although, no structural changes were detected in a supercooled organic propylene glycol liquid using neutron diffraction on cooling [134] some structural changes are found on cooling the Na_2_O⋅B_2_O_3_ one [135]. Metallic glasses, however, exhibit significant structural changes in the supercooled liquid state and such changes are found to be responsible for the liquid fragility. A relatively fragile Pd_42.5_Cu_30_Ni_7.5_P_20_ melt (its fragility index *m* is close to 60 in Table 1) during cooling was studied by using the real-space *PDF*(*R*) function. As a result strong correlation between the change in shape of *PDF*(*R*) function and the variation of viscosity was observed in the supercooled liquid in-situ cooled down to the glass-transition region [136]. The rate of structural change was enhanced in the supercooled liquid towards *T_g_*. Intensification of the covalent bonding between the metallic atoms (especially Ni and Cu) and P found in the Pd-Cu-Ni-P melt and the atomic structure changes of a liquid were responsible for the its fragile behavior because the structural changes induce variation of the activation energy for viscous flow with temperature in Equation (2). 

**Figure 7:**
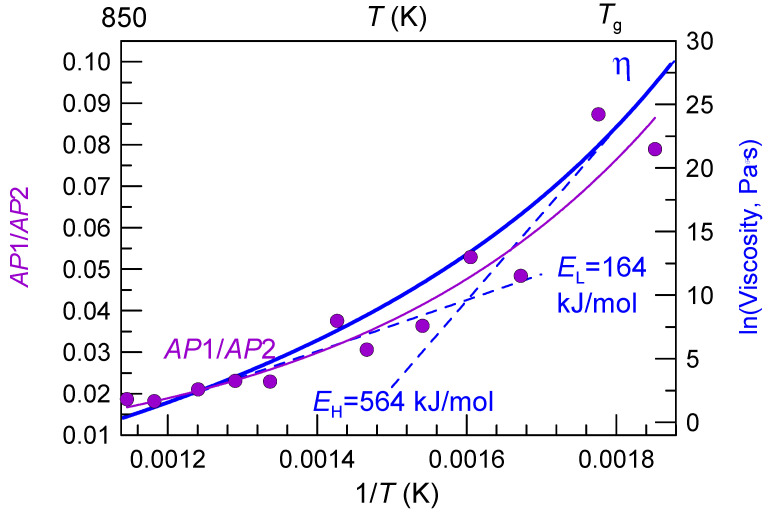
The ratio of the area under the first Cu,Ni-P *PDF*(*R*) peak (*AP*1) and second Pd-Pd *PDF*(*R*) peak (*AP*2) of the Pd_42.5_Cu_30_Ni_7.5_P_20_ glass-forming alloy as a function of inverse temperature (violet circles) and fitting with an exponential growth function y = y_0_ + e^(x–x0/C)^ (violet line) where x is 1/*T*. The data are from Ref. [136]. Solid blue line represents natural logarithm of viscosity as a function of 1/*T* while dashed blue lines represent the activation energies obtained in high (*E*_H_) and low (*E*_L_) temperature areas. The data are taken from Ref. [136].

Figure 7 shows the ratio of the area under the first *PDF*(*R*) peak (*AP*1) related to Cu,Ni-P atomic pairs and second *PDF*(*R*) peak (*AP*2) (Pd-Pd atomic pair) of the Pd_42.5_Cu_30_Ni_7.5_P_20_ glass-forming alloy and ln(*η*), both as a function of inverse temperature. The *AP*1/*AP*2 peak ratio increases nearly 4 times from a relatively low value *AP1/AP2* of ~0.02 at high temperature to 0.08 at the temperature close to *T*_g_. On the other hand, the activation energy for viscous flow (*E*_a_ in Equation (2)) calculated as a tangent to the plot ln(*η*)F(1/*T*) increases from 164 to 564 kJ mol. The ratio of *E*_H_/*E*_L_ of 3.4 is very close to about 4 times increase in *AP1/AP2* within the same temperature interval. This can be taken as an evidence that the structural changes related to intensification of covalent bonds are responsible for the change in *E*_a_ with temperature, and thus fragile behavior of this liquid. 

Strong glass-forming liquids like SiO_2_ show little structural changes on cooling at least in short range order up to about 0.3 nm *R* distance on cooling from 2100 °C [137]. At the same time there are some structural changes at the distances from 0.3 to 1 nm. Here one should note that liquid SiO_2_ starts to evaporate quickly above 1870 °C and it can alter the results [137]. On the other hand a decrease in the position and width of the Si–O *PDF*(*R*) peak was found near *T_g_* [138].

An increase in atomic cooperativity of relaxation (the number of atoms participating in cooperative atomic rearrangements) on cooling was also supposed to be responsible for the liquids fragility as mentioned in Ref. [139]. However, as we know the viscosity as a function of temperature again follows the Arrhenius equation slightly above and below *T_g_* (Figure 4b). This fact can be less easily explained by increase in atomic cooperativity. One can suppose that the rate of collective atomic relaxation (alpha relaxation) without the local atomic structure changes is higher than the rate of those structural changes which are responsible for the fragility. Another reason can be connected with configurons. At the glass transition temperature and below it there are so few configurons (broken bonds with the atomic environment) in the material that for the initiation of the atomic flow it is necessary for the system to create and move them thus leading to high *E*_H_. Opposite to that liquid phase is full of configurons and for the atomic movement it is enough just to move them with low *E*_L_ [140]. It was also suggested [29] that a transition of an atomic system from one local energy landscape minimum to another with temperature depending on the number of atoms involved in the rearrangement is also a signature of liquid fragility.

The changes in electronic structure observed in liquid Si [141] and in a supercooled As_2_Se_3_ liquid on cooling were found to be responsible in transformation of a two-dimensional atomic network structure to a three-dimensional one [142]. In view of this it is important to understand which is the cause and which is the sequence: changes in the electronic structure or the changes in TSRO and CSRO.

It was also found that formation of a heterogeneous microstructure on micrometer scale detected in terms of the absorption coefficient and density was observed in the Pd_40_Cu_30_Ni_10_P_20_ bulk metallic glass [143] though it is not related to chemical phase separation which is found in other glasses owing to repulsive interaction between some constituent elements [144]. The nature of this phenomenon requires further study. 

The fragile melt behavior also was analyzed in reciprocal space. A structural parameter, *γ*, based on the shift of the first peak in the structure factor, *S*(*Q*_1_) is used to characterize the liquid fragility [145]. It was demonstrated for Ni–Nb–Ta [146] and other [147] liquid alloys. Similar structural changes of continuous chemical and topological ordering processes were observed in Zr-Cu [148,149] liquid on cooling. Rather low fragility found for the Zr-Pt metallic melts [147] was explained by localized polar interatomic bonds between Zr and Pt atoms. SRO and MRO develop significantly during cooling the liquid phase to the glassy state in a ternary Zr_60_Cu_30_Al_10_ alloy [150]. 

**Figure 8:**
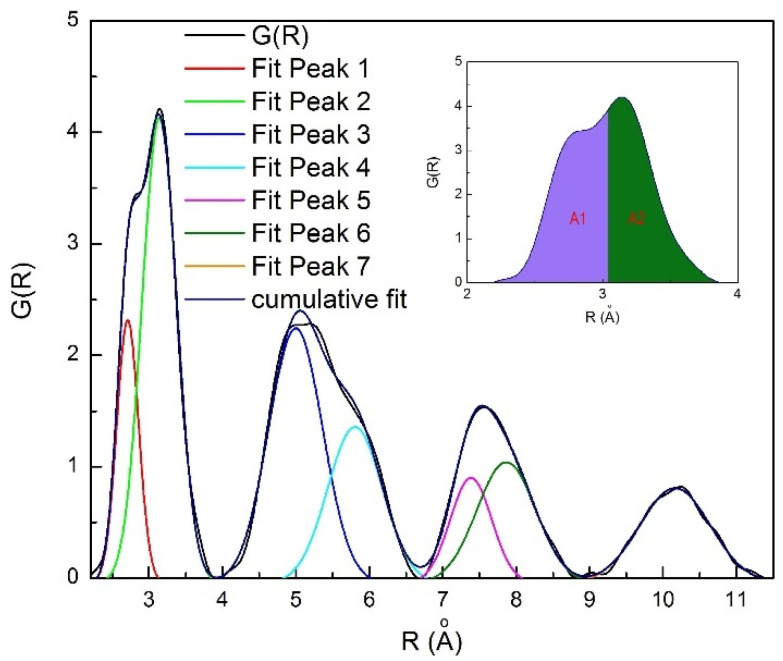
Fitting of four *G(R)* maxima (black curve) of the Zr_55_Cu_30_Ni_5_Al_10_ glass-forming alloy from 0.23 to 1.15 nm obtained at 501 K using seven Gaussian functions related to P1–P7 (as indicated) reproduced from Ref. [152] with permission of Elsevier (copyright year 2020, copyright owner Elsevier). The baseline was subtracted. The value at which two areas A1 and A2 (purple and green) are equal (*R^i^_50_*) is shown in the inset.

The atomic structure changes in a relatively strong Zr_55_Cu_30_Ni_5_Al_10_ glass-forming liquid [151] (the fragility parameter *m* for this liquid is 45, see Table 1) were also monitored by in-situ synchrotron radiation diffractometry. Seven Gaussian function peaks describe well the entire shape of *G(R)* function (oscillating near 0 while *PDF*(R) oscillates near 1) as shown in Figure 8. As in case of the Pd-Cu-Ni-P glass-forming alloy discussed above the first and second *G(R)* maxima of Zr_55_Cu_30_Ni_5_Al_10_ consist of two peaks (P1 and P2), especially at a low temperature [152]. The first peak (P1) in the first coordination shell is related to the nearest Zr-(Cu,Ni) distances. The second peak (P2) mostly corresponds to Zr-Zr pair. Zr-Al interatomic distances are also closer to the value for the second peak position.

**Figure 9:**
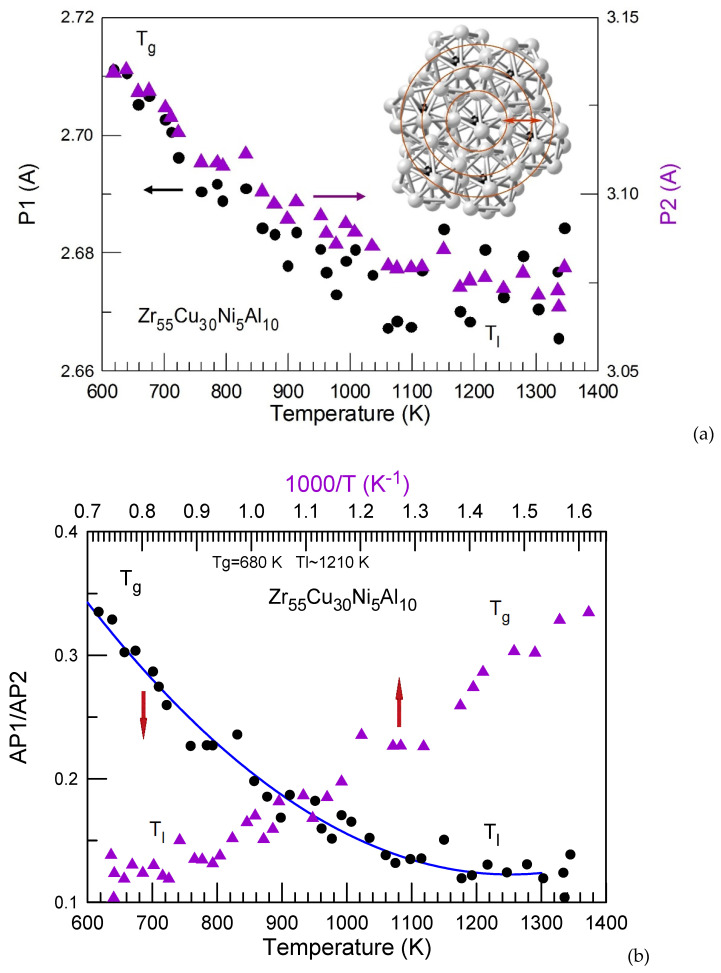

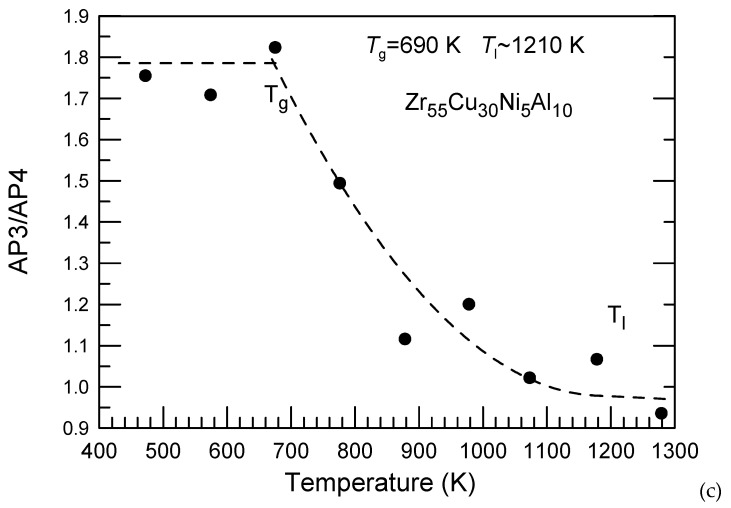
Positions of P1 and P2 in the first coordination shell (**a**) and the ratio of the corresponding peak areas (*AP1/AP2*) (**b**) as a function of temperature (**a**,**b**) and inverse temperature in (**b**). The arrows in (**a**) indicate Y-axes corresponding to the data while the arrows in (**b**) indicate the corresponding X-axes. The insert in (**a**) is a schematic representation of atomic positions within and between the first and higher order coordination shells indicated by red circles. The plots were reproduced from Ref. [152] with permission of Elsevier. The area ratios *AP*3/*AP*4 as a function of temperature (**c**) are calculated from *G*(*R*) functions from the earlier work [152].

As it was also found for the Pd-Cu-Ni-P [19,153], Pd-Si [154,155], Cu-Zr [156], Zr-Cu-Ni-Al [157], Fe-B [37], many other alloys [158] as well as for pure metals [159] opposite to the peaks related to other coordination shells, P1 and P2 of the first coordination shell of the Zr_55_Cu_30_Ni_5_Al_10_ liquid alloy shift to higher values on supercooling below *T*_l_ (Figure 9a). This indicates constant variation of local order in liquids. On cooling, the peak position in the 2nd coordination shell (P3 and P4), in general, did not change with temperature. At the same time contraction in 3rd (P5 and P6) and 4th (P7) coordination shells took place on cooling. The insert in Figure 9a is a schematic representation of atomic redistribution within and between the first and higher order coordination shells. As indicated by the red double side arrow continuous structure changes in the metallic liquids on heating and cooling induce redistribution of the atomic number density.

The ratio of areas under P1 and P2 is nearly constant at high temperature but changes significantly below *T*_l_ (Figure 9b) in line with steep rise of viscosity close [160] to *T_g_*. Similar behavior is observed for P3 and P4 ratio (Figure 9c). P3 becomes stronger on cooling and stops growing below *T*_g_. However, interpretation of these peaks related to the second coordination shell (second red ring in the inset of Figure 9a) is more complicated. An increase in *R^i^_50_* values shown in Figure 8 is found on lowering temperature. The coordination number (CN) in the first coordination shell increases on cooling to a value of 13.3 at 501 K, while at 1335 K CN = 13.0.

The temperature dependence of the first two sub-peaks intensity ratio in the supercooled liquid alloy follows variation of viscosity. It suggests that an increased chemical short range order due to formation of the atomic clusters is directly responsible for the rapid non-Arrhenius increase in viscosity of a supercooled liquid upon cooling. Continuous structural changes in the supercooled liquid leading to the formation of preferred atomic bonds in the first coordination shell and modification of medium range order both change the activation energy (*E_a_*) for viscous flow in Equation (2), and thus, cause deviation from the Arrhenius temperature dependence of viscosity which in term determines fragility of the glass-forming liquids.

As in case of the Pd-Cu-Ni-P alloy [136] *AP1/AP2* (the area under P1 divided by the area under P2) ratio changes on cooling in the supercooled liquid state. However, the changes in *AP1/AP2* ratio for the Zr_55_Cu_30_Ni_5_Al_10_ liquid alloy from *T_l_* to *T_g_* (*AP1/AP2* = 2.5) are smaller in the absolute values compared to those found in the Pd_40_Cu_30_Ni_10_P_20_ liquid alloy for which *AP1/AP2* = 4.5 [136]. The *AP1/AP2* ratio values normalized per Kelvin (*APR^n^*) according to the supercooled liquid region on cooling (*T*_l_–*T*_g_) are 0.005 and 0.017 K^–1^, respectively, are in line with a lower fragility of the Zr_55_Cu_30_Ni_5_Al_10_ melt compared to that of the Pd_40_Cu_30_Ni_10_P_20_ one.

The difference in the specific heat capacity (*C_p_*) at *T_g_* between the liquid and glassy phases is also used to estimate fragility [161]. Δ*C_p_^l–g^* values (difference between *C_p_* of liquid (*C_p_^l^*) and glassy (*C_p_^g^*) phases) of 10 and 17 J/mol⋅K for Zr_55_Cu_30_Ni_5_Al_10_ and Pd_42.5_Cu_30_Ni_7.5_P_20_ alloys, respectively [152], are also in line with the fragility parameters of these alloys. 

**Figure 10:**
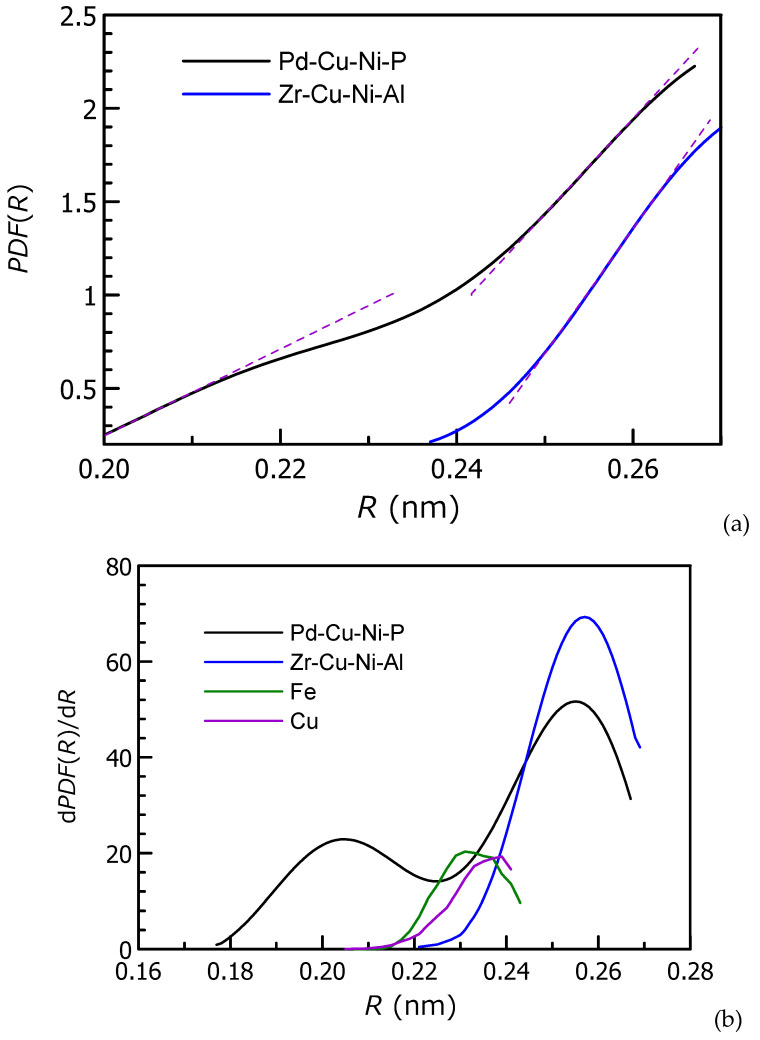
(**a**) *PDF*(*R*) of the Pd_42.5_Cu_30_Ni_7.5_P_20_ and Zr_55_Cu_30_Ni_5_Al_10_ bulk metallic glasses at room temperature. The data were used from Refs. [136,152]. (**b**) d*PDF*(*R*)/d*R* of the Pd_42.5_Cu_30_Ni_7.5_P_20_, Zr_55_Cu_30_Ni_5_Al_10_ (this time *PDF*(*R*) = g(*R*) is used) BMGs bulk metallic glasses as well as of Fe and Cu (MD simulation shown in Figure 2) as a function of *R*.

Metallic glasses exhibiting more fragile supercooled liquid behavior showed a higher degree of density fluctuations probed using synchrotron X-ray nanoscale computed tomography [138,162]. It was also proposed that there is a link between steepness of the repulsive part of the interatomic potential (derived from the shape of *PDF*(*R*)) and rate at which shear modulus decreases with temperature [163]. This mechanism is suggested to control the temperature dependence of viscosity and can lead to fragile behavior of liquids with steep interatomic repulsion while in strong glasses the repulsion is softer. However, a simple shape of *PDF*(*R*) is mostly in pure metals (see Figure 1) while in alloys with different interatomic pairs it becomes more complicated (Figure 10), especially if there is a weak low *R* value peak like in the Pd_42.5_Cu_30_Ni_7.5_P_20_ BMG [136]. The derivative d*PDF*(*R*)/d*R* can also be treated as a parameter indicating steepness of the left shoulder of *PDF*(*R*) peak. Here there is opposite correlation between the value of the derivative and fragility (fragilities of pure metals are considered to be the highest). Zr_55_Cu_30_Ni_5_Al_10_ forms the strongest liquid while the left shoulder of *PDF*(*R*) peak is the steepest.

## 6. Confirmation of the Experimental Results by Classical and Ab-Initio Computer Simulation

As has been shown above the atomic structure of alloys can be modeled using classical and first-principles MD simulation. Classical MD modeling over a million of atoms for simulation time-scales up to hundreds of nanoseconds is especially applicable to model liquids with the relaxation times of tens of picoseconds range. Moreover, when a suitable potential is used the atomic structures of glass-forming liquids are in good agreement with experiment. First-principles/quantum MD operating with the density functional theory is much more precise but can model only up to a thousand of atoms up to picoseconds timescale. The changes in electronic structure of the metallic liquids with temperature are found to be responsible for the atomic clustering in short and medium range, especially in a fragile Pd-Cu-Ni-P system alloy [136].

Classical molecular dynamics simulation suggested that icosahedral ordering in the Cu-Zr glass is an origin of the non-Arrhenius dynamical behavior in metallic supercooled liquids [164,165]. Computer simulations also showed that liquid fragility of metallic glass-forming liquids may be connected with local structure ordering [166]. Structural changes in the Cu-Zr [167,168] and Cu-Zr-Al glasses [169,170] were also studied by MD computer simulation. The dynamic heterogeneities which were discussed above were suggested to be related to a structural heterogeneities caused by a strong interplay between chemical and topological short-range ordering reflected in the temperature dependence of partial pair-distribution functions of Al-Cr alloys [171]. Local ordering in the liquid phase and the rapid development of icosahedral-based medium-range order in the supercooled liquid by the formation of Al-Cr atomic pairs with it believed to explain high fragility of Al-Zn-Cr liquids. It is another evidence that liquid fragility is related to the atomic structure changes on cooling. The question of possible effect of dynamic heterogeneities on liquid fragility is still an open one.

The structures of liquid Au–Si and Au–Ge alloys modeled by the reverse Monte Carlo technique indicated that Si and Ge atoms substitute for Au atoms, and dense liquid is produced on cooling at the eutectic composition [172]. It was suggested that a liquid containing an excess of Si or Ge beyond the eutectic composition may have some empty spaces in the structure.

**Figure 11:**
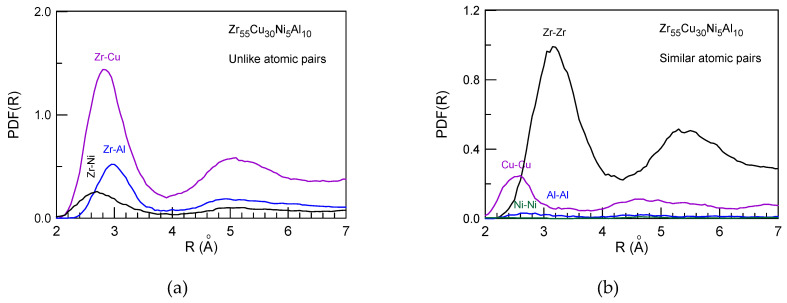

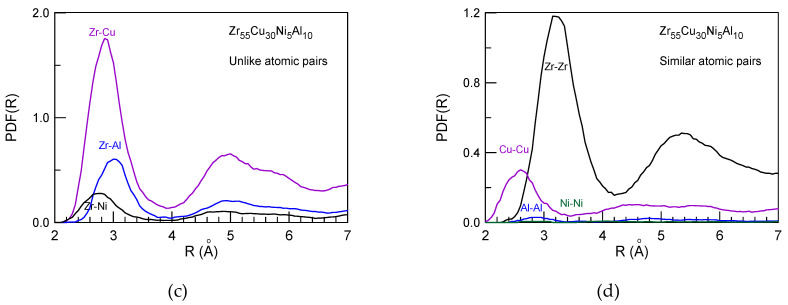
Partial *PDF(R)* functions obtained by computer simulation at 1400 K (liquid state) (**a**,**b**) and 500 K (glass state) (**c**,**d**). Reproduced from Ref. [152] with permission of Elsevier.

*PDF(R)* curves of the Zr_55_Cu_30_Ni_5_Al_10_ glass-forming liquid were also produced by computer simulation. The calculated partial *PDF(R)* functions shown in Figure 11 indicate general ordering and intensification of the Zr-Cu and Zr-Zr sub-peaks on cooling. Moreover, the calculated partial densities of states corresponding to the 3s, 3p, 3d and 4d states of Al, Ni, Cu and Zr atoms, respectively, also indicated some changes in the electronic structure towards a short range order formation in the glass [152]. 

## 7. Conclusive Remarks

The origin of supercooled liquid fragility was a mystery of materials science for many years. From various experimental results there are strong reasons to believe that the origin of fragile behavior is connected with the atomic and electronic structure changes in the supercooled liquids of various metallic glass-forming alloys leading to the variation in the activation energy for viscous flow. The area under the interatomic peak ratio increases nearly in the same way as the activation energy for viscous flow. This can be taken as an evidence that the structural changes are responsible for the change in *E*_a_ with temperature. Thus, fragility is rather a sign of instability of the statistically averaged short and medium range order in fragile liquids upon variation of temperature. The question of what change in the atomic (topological/chemical) or electronic structure is the cause and what is the sequence still requires further study.

Computer simulation results also indicated the structure variation as a function of temperature. The changes in electronic structure of the metallic liquids with temperature and concurrent atomic clustering in short and medium range are found to be responsible for the non-Arrhenius type of temperature dependence of viscosity, especially in the fragile metal-metalloid type Pd-Cu-Ni-P system alloy. The calculated partial densities of states corresponding to the 3s, 3p, 3d and 4d states of Al, Ni, Cu and Zr atoms, respectively, also indicated some changes in the electronic structure towards a short range order formation in the Zr-Cu-Ni-Al metal-metal type metallic glass. Owing to the computational timescales the results of computer simulations are closer to the experimental results for liquids than for glasses. Further correlations between structural ordering and dynamical behavior of liquids can be obtained by these methods. More insights are expected to come from MD results especially with current progress in the computational methods.

Liquid-liquid transitions in deeply supercooled state are only slightly studied, not well understood and require more attention of scientific community. Detailed studies using real space structural functions such as *G*(R) or *PDF*(R) including computer modeling are required to shed light on this behavior.

## Figures and Tables

**Table 1:** Fragility parameters for different substances from Refs. [111,112] rounded to integers.

Alloy	*m*
Au_49_Cu_26.9_Si_16.3_Ag_5.5_Pd_2.3_	46
Cu_47_Ti_34_Zr_11_Ni_8_	47
Fe_67_Mo_6_Ni_3.5_Cr_3.5_P_12_C_5.5_B_2.5_	45
Mg_65_Cu_25_Y_10_	45
Ni_69_Cr_8.5_Nb_3_P_16.5_B_3_	57
Pd_40_Cu_30_Ni_10_P_20_	60
Pt_57.3_Cu_14.6_Ni_5.3_P_22.8_	62
Zr_46.75_Be_27.5_Ti_8.25_Cu_7.5_Ni_10_	44
Zr_55_Cu_30_Ni_5_Al_10_	45
SiO_2_	20
o-terphenyl (organic liquid)	81
n-propanol	35
poly(ethylene oxide)	23

## Data Availability

The data can be provided upon request.
